# Quantum Codes from Galois Hulls of Constacyclic Codes over a Finite Non-Chain Ring

**DOI:** 10.3390/e28040407

**Published:** 2026-04-03

**Authors:** Enbin Zhang, Bo Kong, Xiying Zheng

**Affiliations:** 1 School of Statistics and Mathematics, Henan Finance University, Zhengzhou 450046, China; zhangenbin@hafu.edu.cn; 2 Faculty of Engineering, Huanghe Science and Technology College, Zhengzhou 450063, China

**Keywords:** quantum code, constacyclic code, Galois hull, construction X

## Abstract

We study the algebraic structures of constacyclic codes over a new finite non-chain ring R and their *l*-Galois dual codes based on a new Gray map ϕ and determine the dimensions of the *l*-Galois hull of constacyclic codes over R. Furthermore, we propose a quantum construction X of the Galois inner product to construct quantum codes by combining the Calderbank–Shor–Steane (CSS) construction, Hermitian construction and construction X. As an application, by calculating the dimensions of the *l*-Galois hull of constacyclic codes over R, we obtain some new quantum maximum distance separable (QMDS) codes and new quantum error-correcting codes (QECCs) that are better than the best known ones.

## 1. Introduction

Quantum error-correcting code (QECC) is an effective coding scheme for quantum communication and quantum computation. Shor [1] constructed the world’s first QECC [[9,1,3]] in 1995. In 1996, Calderbank and Shor [2] and Steane [3] introduced a systematic and effective method for constructing QECCs by using two special classical binary error-correcting codes, respectively, which is called the CSS construction method. In 1998, Calderbank et al. [4] proposed a Hermitian construction method to effectively transform the problem of error-correcting code in a finite field $F_{4}$. These constructions need to find linear codes that satisfy the Euclidean dual-containing or Hermitian dual-containing condition with respect to the Euclidean inner product or Hermitian inner product. Sometimes, the dual-containing condition is difficult to satisfy. To solve this problem, Brun et al. [5] proposed an entanglement-assisted quantum error-correcting code (EAQECC) using the classical linear codes without the dual-containing condition. Some entanglement-assisted quantum maximum distance separable (EAQMDS) codes have been constructed by studying the algebraic structure of constacyclic codes [6,7]. Lin et al. [8] constructed EAQECCs from Gray images of constacyclic codes over a finite non-chain ring. Since Sangwisut et al. [9] studied the hulls of cyclic codes with respect to the Euclidean inner product and Hermitian inner product over a finite field, some EAQECCs were obtained by determining the dimension of the Euclidean hull or Hermitian hull of cyclic codes [10,11] or constacyclic codes [12].

The Galois hull of constacyclic codes is a generalization of the Euclidean hull and Hermitian hull. It can be used to construct EAQECCs by determining the dimension of the Galois hull. Fan and zhang [13] introduced the Galois inner product by generalizing the Euclidean inner product and Hermitian inner product and studied Galois self-dual constacyclic codes over finite fields. Liu et al. [14] studied the Galois linear complementary dual (LCD) codes over finite fields. Liu and Pan [15] studied the Galois hull of linear codes over a finite field. Cao [16] and Li [17] obtained EAQECCs from maximum distance separable (MDS) codes with a Galois hull of arbitrary dimensions. Li and Zhu [18] constructed EAQECCs from linear codes with a Galois hull of arbitrary dimensions from Galois self-orthogonal codes.

In 2014, quantum construction X, a composite method, was proposed by Lison$\overset{ˇ}{e}$k and Singh [19]. It was used to construct QECCs based on the Hermitian dual or Euclidean dual condition of linear codes over $F_{4}$. Degwekar et al. [20] extended the quantum construction X from $F_{4}$ to $F_{p^{2}}$ and obtained some QECCs, where *p* is a prime. Ezerman et al. [21] constructed QECCs from quasi-cyclic codes with large Hermitian hulls over $F_{4}$ and $F_{9}$ by applying the quantum construction X. Zhang [22] established three methods of constructing quantum QECCs via the quantum construction X of the Euclidean dual condition over finite fields. Hu and Liu [23] obtained QECCs using the quantum construction X by determining the dimensions of the hulls of the cyclic codes. Tian et al. [24] constructed QECCs through quantum construction X by studying the dimensions of hulls of constacyclic codes over finite non-chain rings.

Constructing EAQECCs needs to determine the dimensions of Galois hulls, and constructing QECCs needs to find linear codes that satisfy the dual-containing conditions. However, the dimensions of the Galois hull of constacyclic codes are difficult to determine, and the dual-containing conditions are difficult to satisfy. Due to the strong motivation discussed above, we construct EAQECCs and QECCs by determining the dimensions of the *l*-Galois hull of constacyclic codes. In Section 2, we define a new finite non-chain ring R and a Gray map ϕ, and give some basic concepts. Some results on constacyclic codes and their *l*-Galois dual codes are provided in Section 3. We present some results on the *l*-Galois hull of constacyclic codes in Section 4. In Section 5, we study the dimensions of the *l*-Galois hulls of the Gray images of constacyclic codes and construct EAQECCs and QECCs.

## 2. Preliminaries

In this section, we will define a new finite non-chain ring R and a Gray map ϕ, recall some basic concepts and properties, and extend them to R.

Let

$$R=F_{p^{m}}\left[u_{1},u_{2},\cdots ,u_{s}\right]/\langle f_{1}\left(u_{1}\right),\cdots ,f_{s}\left(u_{s}\right),u_{i}u_{j}=u_{j}u_{i}=0\rangle ,$$

where $F_{p^{m}}$ is the finite field with size $p^{m}$, $f_{j}\left(u_{j}\right)=u_{j}^{l_{j}}-u_{j}=\left(u_{j}-\alpha_{j,1}\right)\left(u_{j}-\alpha_{j,2}\right)\cdots \left(u_{j}-\alpha_{j,l_{j}}\right)$, *p* is a prime number, *m* and $l_{j}$ are positive integers, $l_{j}\geq 2$, i,j=1,2,⋯,s.

Let

$$\varpi_{ij}=\frac{\left(u_{j}-\alpha_{j,1}\right)\cdots \left(u_{j}-\alpha_{j,i-1}\right)\left(u_{j}-\alpha_{j,i+1}\right)\cdots \left(u_{j}-\alpha_{j,l_{j}}\right)}{\left(\alpha_{j,i}-\alpha_{j,1}\right)\cdots \left(\alpha_{j,i}-\alpha_{j,i-1}\right)\left(\alpha_{j,i}-\alpha_{j,i+1}\right)\cdots \left(\alpha_{j,i}-\alpha_{j,l_{j}}\right)},$$

we have $\varpi_{ij}^{2}=\varpi_{ij}$, and $\varpi_{ij}\varpi_{i^{\prime }j^{\prime }}=0$ when $\left(i,j\right)\neq \left(i^{\prime },j^{\prime }\right)$, where $2\leq i\leq l_{j},1\leq j\leq s$.

For simplicity, in this paper, let $t=l_{1}+\cdots +l_{s}-s+1$.

Let $e_{1}=\varpi_{21},e_{2}=\varpi_{22},\cdots ,e_{s}=\varpi_{2s},\cdots ,e_{t-1}=\varpi_{l_{s}s},e_{t}=1-\Sigma_{ij}\varpi_{ij}$, then $e_{i}^{2}=e_{i}$ and $e_{i}e_{j}=0$ for i≠j.

Thus $e_{1}+e_{2}+\cdots +e_{t}=1$, where i,j=1,2,⋯,t.

By the Chinese Remainder Theorem, we have $R=e_{1}R⊕e_{2}R⊕\cdots ⊕e_{t}R=e_{1}F_{p^{m}}⊕e_{2}F_{p^{m}}⊕\cdots ⊕e_{t}F_{p^{m}}$.

For any r∈R, there exists a unique expression of the form $r=r_{1}e_{1}+r_{2}e_{2}+\cdots +r_{t}e_{t}$, where $r_{i}\in F_{p^{m}}$ for i=1,2,⋯,t.

Let σ be the Frobenius automorphism of $F_{p^{m}}$, which is defined as

$$\begin{array}{c} \begin{array}{cc} \sigma :\;\; & F_{p^{m}}\to F_{p^{m}},\\ & a\mapsto a^{p}. \end{array} \end{array}$$

For any $x=\left(x_{1},x_{2},\cdots ,x_{n}\right)\in F_{p^{m}}^{n}$ and any matrix $G={\left(m_{i,j}\right)}_{s\times t}$ over $F_{p^{m}}$, we denote $\sigma \left(x\right)=(\sigma \left(x_{1}\right),\sigma \left(x_{2}\right),\cdots ,\sigma \left(x_{n}\right))$ and $\sigma \left(G\right)={\left(\sigma \left(m_{i,j}\right)\right)}_{s\times t}$. It is easy to see that σ is a bijective map from $F_{p^{m}}^{n}$ to $F_{p^{m}}^{n}$.

Then we extend σ from R to R as follows.

$$\begin{array}{c} \begin{array}{cc} \sigma :\; & R\to R,\\ & r=e_{1}r_{1}+e_{2}r_{2}+\cdots +e_{t}r_{t}\mapsto r^{p}=e_{1}r_{1}^{p}+e_{2}r_{2}^{p}+\cdots +e_{t}r_{t}^{p}, \end{array} \end{array}$$

it is easy to see that σ is an automorphism of R.

Similarly, for any $x=\left(x_{1},x_{2},\cdots ,x_{n}\right)\in R^{n}$ and any matrix $G={\left(m_{i,j}\right)}_{s\times t}$ over R, we denote $\sigma \left(x\right)=(\sigma \left(x_{1}\right),\sigma \left(x_{2}\right),\cdots ,\sigma \left(x_{n}\right))$ and $\sigma \left(G\right)={\left(\sigma \left(m_{i,j}\right)\right)}_{s\times t}$.

**Definition** **1** ([13])**.** *Let $x=(x_{1},x_{2},\cdots ,x_{n})$ and $y=\left(y_{1},y_{2},\cdots ,y_{n}\right)\in F_{p^{m}}^{n}$, for each integer l with 0≤l≤m−1, the l-Galois inner product of x,y is defined as*

$${\langle x,y\rangle }_{l}=\sum_{i=1}^{n}x_{i}y_{i}^{p^{l}}.$$

Clearly, when l=0, the *l*-Galois inner product is the Euclidean inner product; when *m* is even and $l=\frac{m}{2}$, the *l*-Galois inner product is the Hermitian inner product.

When ${\langle x,y\rangle }_{l}=0$, the vectors x and y are said to be *l*-Galois orthogonal.

**Definition** **2** ([13])**.** *For any code C over $F_{p^{m}}$, its l-Galois dual code is defined as*

$$C^{{⊥}_{l}}=\left\{x|{\langle y,x\rangle }_{l}=0,\forall \;y\in C\right\}.$$

Then we extend Definitions 1 and 2 from $F_{p^{m}}$ to R, and we can obtain the following definitions.

**Definition** **3.** *Let $x=(x_{1},x_{2},\cdots ,x_{n})$ and $y=(y_{1},y_{2},\cdots ,y_{n})\in R$, for each integer l with 0≤l≤m−1, the inner product*

$${\langle x,y\rangle }_{l}=\sum_{i=1}^{n}x_{i}y_{i}^{p^{l}}.$$

*is called the l-Galois inner product of x,y.*

**Definition** **4.** *Let C be a linear code of length n over R, the code*

$$C^{{⊥}_{l}}=\left\{x|{\langle y,x\rangle }_{l}=0,\forall \;y\in C\right\}.$$

*is called the l-Galois dual code of C.*

Then, $C^{{⊥}_{0}}$ and $C^{{⊥}_{\frac{l}{2}}}$ represent the Euclidean dual code and the Hermitian dual code of *C*, respectively.

If $C\subseteq C^{{⊥}_{l}}$, then *C* is *l*-Galois self-orthogonal; if $C^{{⊥}_{l}}\subseteq C$, then *C* is *l*-Galois dual-containing; and if $C=C^{{⊥}_{l}}$, then *C* is *l*-Galois self-dual.

**Definition** **5.** *The l-Galois hull of C is defined by ${\mathrm{Hull}}_{l}\left(C\right)=C\cap C^{{⊥}_{l}}$. Then C is an l-Galois self-orthogonal code if ${\mathrm{Hull}}_{l}\left(C\right)=C$, and it is an l-Galois dual-containing code if ${\mathrm{Hull}}_{l}\left(C\right)=C^{{⊥}_{l}}$.*

**Definition** **6** ([9])**.** *Let C be a linear code of length n over R and λ be a unit of R. If for any $c=(c_{1},c_{2},\cdots ,c_{n})\in C$ implying $\delta_{\lambda }\left(c\right)=\left(\lambda c_{n},c_{1},\cdots ,c_{n-1}\right)\in C$, then C is called a λ-constacyclic code of length n over R. In particular, when λ=−1, C is called a negacyclic code, and when λ=1, C is called a cyclic code.*

Let $f\left(x\right)=a_{0}+a_{1}x+a_{2}x^{2}+\cdots +a_{r-1}x^{r-1}+a_{r}x^{r}$ be a polynomial with $a_{0}\neq 0$. Then the reciprocal polynomial of f(x) is denoted by $f^{*}\left(x\right)=a_{0}^{-1}x^{r}f\left(x^{-1}\right)$.

Define a Gray map $\phi :\;R\to F_{p^{m}}^{t}$ as $r=e_{1}r_{1}+e_{2}r_{2}+\cdots +e_{t}r_{t}\mapsto \left(r_{1},r_{2},\cdots ,r_{t}\right)$, and we extend ϕ as

$$\begin{array}{c} \begin{array}{cc} \phi :\;\; & R^{n}\to F_{p^{m}}^{tn},\\ & \left(r_{1},r_{2},\cdots ,r_{n}\right)\mapsto \left(r_{1,1},\cdots ,r_{1,t},r_{2,1},\cdots ,r_{2,t},\cdots ,r_{n,1},\cdots ,r_{n,t}\right), \end{array} \end{array}$$

where $r_{i}=r_{i,1}e_{1}+r_{i,2}e_{2}+\cdots +r_{i,t}e_{t}\in R$ for i=1,2,⋯,n.

For any r∈R, we define the Gray weight of *r* as $w_{G}\left(r\right)=w_{H}\left(\phi \left(r\right)\right)$, where $w_{H}\left(\phi \left(r\right)\right)$ is the Hamming weight of ϕ(r).

For any $x=(x_{1},x_{2},\cdots ,x_{n})$, $y=\left(y_{1},y_{2},\cdots ,y_{n}\right)\in R^{n}$, the Gray distance of x,y is defined as $d_{G}\left(x,y\right)=w_{G}\left(x-y\right)$, the Gray distance of *C* is defined as $d_{G}\left(C\right)=\min\left\{d_{G}\left(x,y\right)|x,y\in C,x\neq y\right\}$, and $w_{G}\left(x-y\right)$ is the Gray weight of x−y, defined as $w_{G}\left(x-y\right)=\sum_{i=1}^{n}w_{G}\left(x_{i}-y_{i}\right)$.

Let *C* be a linear code of length *n* over R. Let

$$\begin{array}{c} \begin{array}{cc} & C_{1}=\left\{r_{1}\in F_{p^{m}}^{n}|e_{1}r_{1}+e_{2}r_{2}+\cdots +e_{t}r_{t}\in C,\exists \;r_{2},r_{3},\cdots ,r_{t}\in F_{p^{m}}^{n}\right\},\\ & C_{2}=\left\{r_{2}\in F_{p^{m}}^{n}|e_{1}r_{1}+e_{2}r_{2}+\cdots +e_{t}r_{t}\in C,\exists \;r_{1},r_{3},\cdots ,r_{t}\in F_{p^{m}}^{n}\right\},\\ & \cdots ,\\ & C_{t}=\left\{r_{t}\in F_{p^{m}}^{n}|e_{1}r_{1}+e_{2}r_{2}+\cdots +e_{t}r_{t}\in C,\exists \;r_{1},r_{2},\cdots ,r_{t-1}\in F_{p^{m}}^{n}\right\}. \end{array} \end{array}$$

Clearly, $C_{i}$ is a linear code of length *n* over $F_{p^{m}}$ for i=1,2,⋯,t. So,

$$\begin{array}{c} C=e_{1}C_{1}⊕e_{2}C_{2}⊕\cdots ⊕e_{t}C_{t} \end{array}$$

and

$$\begin{array}{c} |C|=|C_{1}||C_{2}|\cdots |C_{t}|. \end{array}$$

Let $G_{i}$ be a generator matrix of $C_{i}$ for i=1,2,⋯,t, then the dimension of *C* is

$$\begin{array}{c} \dim\left(C\right)=\dim\left(C_{1}\right)+\dim\left(C_{2}\right)+\cdots +\dim\left(C_{t}\right) \end{array}$$

and the generator matrix of *C* is

$$\begin{array}{c} G=\left(\begin{array}{c} e_{1}G_{1}\\ e_{2}G_{2}\\ \cdots \\ e_{t}G_{t} \end{array}\right). \end{array}$$

By the same method of Lemma 3.1 in [25], we can have the following lemma.

**Lemma** **1.** *$(\lambda_{1}e_{1}+\lambda_{2}e_{2}+\cdots +\lambda_{t}e_{t})\in R$ is a unit in R if and only if $\lambda_{1},\lambda_{2},\cdots ,\lambda_{t}$ are units in $F_{p^{m}}$.*

By the same method of Lemma 1 in [26], we can have the following lemma.

**Lemma** **2.** *ϕ is both a bijection and a distance preserving map from $R^{n}$ to $F_{p^{m}}^{tn}$.*

**Lemma** **3** ([15])**.** *Let C be a linear code of length n over $F_{p^{m}}$. For any 0≤l≤m−1, then*

*(1)*  *$C^{{⊥}_{l}}=\sigma^{m-l}\left(C^{{⊥}_{0}}\right)$.*
*(2)*  *${\left(C^{{⊥}_{l}}\right)}^{{⊥}_{f}}=\sigma^{2m-l-f}\left(C\right)$, for any 0≤l,f≤m−1. In particular, ${\left(C^{{⊥}_{0}}\right)}^{{⊥}_{0}}=C$, and ${\left(C^{{⊥}_{\frac{m}{2}}}\right)}^{\frac{m}{2}}=C$ if m is even.*

## 3. Constacyclic Codes over R

In this section, we will study the algebraic structures of constacyclic codes over R and their *l*-Galois dual codes.

**Lemma** **4** ([26])**.** *Let $C=e_{1}C_{1}⊕e_{2}C_{2}⊕\cdots ⊕e_{t}C_{t}$ be a λ-constacyclic code of length n over R if and only if $C_{j}$ is a $\lambda_{j}$-constacyclic code of length n over $F_{p^{m}}$ for j=1,2,⋯,t, where $\lambda =e_{1}\lambda_{1}+e_{2}\lambda_{2}+\cdots +e_{t}\lambda_{t}$ be a unit in R.*

**Lemma** **5** ([27])**.** *Let R be a finite commutative Frobenius ring with identity, $\sigma ,\tilde{\sigma }\in \mathrm{Aut}\left(R\right)$; and let C be a linear code of length n over R. Then, the following apply:*

*(1)*  *$C^{{⊥}_{\sigma }}$ is a linear code over R.*
*(2)*  *$C^{{⊥}_{\sigma }}=\sigma^{-1}\left(C^{⊥}\right)$ and $|C||C^{{⊥}_{\sigma }}|=|R{|}^{n}$.*
*(3)*  *${\left(C^{{⊥}_{\sigma }}\right)}^{{⊥}_{\tilde{\sigma }}}={\tilde{\sigma }}^{-1}\sigma^{-1}\left(C\right)$.*

**Theorem** **1.** *Let $C=e_{1}C_{1}⊕e_{2}C_{2}⊕\cdots +⊕e_{t}C_{t}$ be a linear code over R. Then*

$$\begin{array}{c} C^{{⊥}_{l}}=e_{1}C_{1}^{{⊥}_{l}}⊕e_{2}C_{2}^{{⊥}_{l}}⊕\cdots ⊕e_{t}C_{t}^{{⊥}_{l}}, \end{array}$$

*where $C_{i}^{{⊥}_{l}}$ is the l-Galois dual code of $C_{i}$ for i=1,2,⋯,t.*

**Proof.**  Let

$$D=e_{1}C_{1}^{{⊥}_{l}}⊕e_{2}C_{2}^{{⊥}_{l}}⊕\cdots ⊕e_{t}C_{t}^{{⊥}_{l}}.$$

For any

$$x=e_{1}x_{1}+e_{2}x_{2}+\cdots +e_{t}x_{t}\in C$$

and any

$$x^{\prime }=e_{1}x_{1}^{\prime }+e_{2}x_{2}^{\prime }+\cdots +e_{t}x_{t}^{\prime }\in D,$$

where $x_{i}=\left(x_{i1},x_{i2},\cdots ,x_{in}\right)\in C_{i}$ and $x_{i}^{\prime }=\left(x_{i1}^{\prime },x_{i2}^{\prime },\cdots ,x_{in}^{\prime }\right)\in C_{i}^{{⊥}_{l}}$, we have

$$\begin{array}{c} {\langle x_{i},x_{i}^{\prime }\rangle }_{l}=\sum_{j=1}^{n}x_{ij}{\left(x_{ij}^{\prime }\right)}^{p^{l}}=0, \end{array}$$

where i=1,2,⋯,t.

Then

$$\begin{array}{c} x=\left(\sum_{i=1}^{t}e_{i}x_{i1},\sum_{i=1}^{t}e_{i}x_{i2},\cdots ,\sum_{i=1}^{t}e_{i}x_{in}\right), \end{array}$$

$$\begin{array}{c} x^{\prime }=\left(\sum_{i=1}^{t}e_{i}x_{i1}^{\prime },\sum_{i=1}^{t}e_{i}x_{i2}^{\prime },\cdots ,\sum_{i=1}^{t}e_{i}x_{in}^{\prime }\right) \end{array}$$

and

$$\begin{array}{c} {\langle x,x^{\prime }\rangle }_{l}=\sum_{j=1}^{n}\left(\sum_{i=1}^{t}e_{i}x_{ij}\right){\left(\sum_{i=1}^{t}e_{i}x_{ij}^{\prime }\right)}^{p^{l}}=\sum_{i=1}^{t}e_{i}\left(\sum_{j=1}^{n}x_{ij}{\left(x_{ij}^{\prime }\right)}^{p^{l}}\right)=0. \end{array}$$

Thus

$$x^{\prime }\in C^{{⊥}_{l}}.$$

Hence

$$\begin{array}{c} D\subseteq C^{{⊥}_{l}}. \end{array}$$

By Lemma 3,

$$C_{i}^{{⊥}_{l}}=\sigma^{m-l}\left(C_{i}^{⊥}\right).$$

Since σ is a bijective map from $F_{p^{m}}^{n}$ to $F_{p^{m}}^{n}$, we have

$$\begin{array}{c} |C_{i}^{{⊥}_{l}}|=|\sigma^{m-l}\left(C_{i}^{⊥}\right)|=|C_{i}^{⊥}|=\frac{p^{mn}}{|C_{i}|}. \end{array}$$

By Lemma 5, we have

$$|C||C^{{⊥}_{\sigma }}|=|R{|}^{n}$$

and

$$C^{{⊥}_{\sigma }}=\sigma^{-1}\left(C^{⊥}\right).$$

Since σ is a bijective map from $R^{n}$ to $R^{n}$, we have

$$\begin{array}{c} |C^{{⊥}_{\sigma }}|=|\sigma^{-1}\left(C^{⊥}\right)|=|C^{⊥}|. \end{array}$$

So

$$\begin{array}{c} |C||C^{{⊥}_{l}}|=|R{|}^{n}. \end{array}$$

Thus,

$$\begin{array}{cc} |D| & =|C_{1}^{{⊥}_{l}}||C_{2}^{{⊥}_{l}}|\cdots |C_{t}^{{⊥}_{l}}|=\frac{p^{mn}}{|C_{1}|}\frac{p^{mn}}{|C_{2}|}\cdots \frac{p^{mn}}{|C_{t}|}=\frac{|R{|}^{n}}{|C|}=|C^{{⊥}_{l}}|. \end{array}$$

Hence,

$$\begin{array}{c} C^{{⊥}_{l}}=D=e_{1}C_{1}^{{⊥}_{l}}⊕e_{2}C_{2}^{{⊥}_{l}}⊕\cdots ⊕e_{t}C_{t}^{{⊥}_{l}}. \end{array}$$

□

**Theorem** **2.** *Let $C=e_{1}C_{1}⊕e_{2}C_{2}⊕\cdots ⊕e_{t}C_{t}$ be a λ-constacyclic code over R, where $\lambda =e_{1}\lambda_{1}+e_{2}\lambda_{2}+\cdots +e_{t}\lambda_{t}$. Then $C^{{⊥}_{l}}=e_{1}C_{1}^{{⊥}_{l}}⊕e_{2}C_{2}^{{⊥}_{l}}⊕\cdots ⊕e_{t}C_{t}^{{⊥}_{l}}$ is a ${\left(\lambda \right)}^{-p^{m-l}}$-constacyclic code of length n over R, where $C_{i}^{{⊥}_{l}}$ is a ${\left(\lambda_{i}\right)}^{-p^{m-l}}$-constacyclic code over $F_{p^{m}}$ for i=1,2,⋯,t.*

**Proof.**  For any $\;a=\left(a_{1},a_{2},\cdots ,a_{n}\right)\in C^{{⊥}_{l}}$ and any $\;b=(b_{1},b_{2},\cdots ,b_{n})\in C$, we have

$$\begin{array}{c} \begin{array}{cc} 0 & ={\langle \delta_{\lambda }^{n-1}\left(b\right),a\rangle }_{l}\\ & =\lambda b_{2}a_{1}^{p^{l}}+\lambda b_{3}a_{2}^{p^{l}}+\cdots +\lambda b_{n}a_{n-1}^{p^{l}}+b_{1}a_{n}^{p^{l}}\\ & =\lambda (b_{2}a_{1}^{p^{l}}+b_{3}a_{2}^{p^{l}}+\cdots +b_{n}a_{n-1}^{p^{l}}+\lambda^{-1}b_{1}a_{n}^{p^{l}}). \end{array} \end{array}$$

It is easy to see that

$$\begin{array}{c} \lambda^{-1}=e_{1}\lambda_{1}^{-1}+e_{2}\lambda_{2}^{-1}+\cdots +e_{t}\lambda_{t}^{-1} \end{array}$$

and

$$\begin{array}{c} \begin{array}{cc} {\left(\lambda \right)}^{-p^{m-l}} & =e_{1}{\left(\lambda_{1}^{-1}\right)}^{p^{m-l}}+e_{2}{\left(\lambda_{2}^{-1}\right)}^{p^{m-l}}+\cdots +e_{t}{\left(\lambda_{t}^{-1}\right)}^{p^{m-l}}\\ & =e_{1}{\left(\lambda_{1}\right)}^{-p^{m-l}}+e_{2}{\left(\lambda_{2}\right)}^{-p^{m-l}}+\cdots +e_{t}{\left(\lambda_{t}\right)}^{-p^{m-l}}. \end{array} \end{array}$$

This implies

$$\begin{array}{c} \lambda^{-1}b_{1}a_{n}^{p^{l}}+b_{2}a_{1}^{p^{l}}+b_{3}a_{2}^{p^{l}}+\cdots +b_{n}a_{n-1}^{p^{l}}=0. \end{array}$$

Hence,

$$\begin{array}{c} {\langle b,\delta_{{\left(\lambda \right)}^{-p^{m-l}}}\left(a\right)\rangle }_{l}=\lambda^{-1}b_{1}a_{n}^{p^{l}}+b_{2}a_{1}^{p^{l}}+b_{3}a_{2}^{p^{l}}+\cdots +b_{n}a_{n-1}^{p^{l}}=0. \end{array}$$

Thus

$$\begin{array}{c} \delta_{{\left(\lambda \right)}^{-p^{m-l}}}\left(a\right)\in C^{{⊥}_{l}}, \end{array}$$

which implies that $C^{{⊥}_{l}}$ is a ${\left(\lambda \right)}^{-p^{m-l}}$-constacyclic code.

Hence $C^{{⊥}_{l}}$ is an $\left(e_{1}{\left(\lambda_{1}\right)}^{-p^{m-l}}+e_{2}{\left(\lambda_{2}\right)}^{-p^{m-l}}+\cdots +e_{t}{\left(\lambda_{t}\right)}^{-p^{m-l}}\right)$-constacyclic code of length *n* over R.

By Theorem 1 and Lemma 4, $C_{i}^{{⊥}_{l}}$ is a ${\left(\lambda_{i}\right)}^{-p^{m-l}}$-constacyclic code over $F_{p^{m}}$ for i=1,2,⋯,t. □

By the same method of Theorem 2 in [26], we can have the following theorem.

**Theorem** **3.** *Let $C=e_{1}C_{1}⊕e_{2}C_{2}⊕\cdots ⊕e_{t}C_{t}$ be an $\left(e_{1}\lambda_{1}+e_{2}\lambda_{2}+\cdots +e_{t}\lambda_{t}\right)$-constacyclic code of length n over R. Then $C=\langle e_{1}g_{1}\left(x\right)+e_{2}g_{2}\left(x\right)+\cdots +e_{t}g_{t}\left(x\right)\rangle$, where $g_{i}\left(x\right)\in F_{p^{m}}\left[x\right]$ is a generator polynomial of the $\lambda_{i}$-constacyclic $C_{i}$ for i=1,2,⋯,t.*

**Corollary** **1.** Let $C=e_{1}C_{1}⊕e_{2}C_{2}⊕\cdots ⊕e_{t}C_{t}$ be an $\left(e_{1}\lambda_{1}+e_{2}\lambda_{2}+\cdots +e_{t}\lambda_{t}\right)$-constacyclic code of length n over R. Then $C^{{⊥}_{l}}=\langle e_{1}\sigma^{m-l}\left(f_{1}^{*}\left(x\right)\right)+e_{2}\sigma^{m-l}\left(f_{2}^{*}\left(x\right)\right)+\cdots +e_{t}\sigma^{m-l}\left(f_{t}^{*}\left(x\right)\right)\rangle$, where $f_{i}^{*}\left(x\right)$ is the reciprocal polynomial of $f_{i}\left(x\right)=\left(x^{n}-\lambda_{i}\right)/g_{i}\left(x\right)$, defined as $f_{i}^{*}\left(x\right)=a_{i0}^{-1}x^{\deg(f_{i})}f_{i}\left(x^{-1}\right)$, with $a_{i0}$ being the non-zero constant term of $f_{i}\left(x\right)$, i=1,2,⋯,t.

**Proof.**  By the same method of Theorem 4 in [26], we have

$$C_{i}^{⊥}=\langle f_{i}^{*}\left(x\right)\rangle$$

for i=1,2,⋯,t.

By Lemma 3,

$$\begin{array}{c} C_{i}^{{⊥}_{l}}={\left(\sigma \right)}^{m-l}\left(C_{i}^{⊥}\right)=\langle \sigma^{m-l}\left(f_{i}^{*}\left(x\right)\right)\rangle . \end{array}$$

By Theorem 1,

$$\begin{array}{c} C^{{⊥}_{l}}=e_{1}C_{1}^{{⊥}_{l}}⊕e_{2}C_{2}^{{⊥}_{l}}⊕\cdots ⊕e_{t}C_{t}^{{⊥}_{l}}. \end{array}$$

Thus,

$$\begin{array}{c} C^{{⊥}_{l}}=\langle e_{1}\sigma^{m-l}\left(f_{1}^{*}\left(x\right)\right),e_{2}\sigma^{m-l}\left(f_{2}^{*}\left(x\right)\right),\cdots ,e_{t}\sigma^{m-l}\left(f_{t}^{*}\left(x\right)\right)\rangle . \end{array}$$

Let

$$\begin{array}{c} D=\langle e_{1}\sigma^{m-l}\left(f_{1}^{*}\left(x\right)\right)+e_{2}\sigma^{m-l}\left(f_{2}^{*}\left(x\right)\right)+\cdots +e_{t}\sigma^{m-l}\left(f_{t}^{*}\left(x\right)\right)\rangle , \end{array}$$

which implies that,

$$\begin{array}{c} D\subseteq C^{{⊥}_{l}}. \end{array}$$

We have

$$\begin{array}{c} e_{i}\left(e_{1}\sigma^{m-l}\left(f_{1}^{*}\left(x\right)\right)+e_{2}\sigma^{m-l}\left(f_{2}^{*}\left(x\right)\right)+\cdots +e_{t}\sigma^{m-l}\left(f_{t}^{*}\left(x\right)\right)\right)=e_{i}\sigma^{m-l}\left(f_{i}^{*}\left(x\right)\right). \end{array}$$

Hence,

$$\begin{array}{c} C^{{⊥}_{l}}\subseteq D \end{array}$$

and

$$\begin{array}{c} C^{{⊥}_{l}}=D=\langle e_{1}\sigma^{m-l}\left(f_{1}^{*}\left(x\right)\right)+e_{2}\sigma^{m-l}\left(f_{2}^{*}\left(x\right)\right)+\cdots +e_{t}\sigma^{m-l}\left(f_{t}^{*}\left(x\right)\right)\rangle . \end{array}$$

□

## 4. *l*-Galois Hull of Constacyclic Codes

In this section, we will determine the dimensions of the *l*-Galois hull of constacyclic codes over R.

**Lemma** **6** ([15]). *Let C be a linear code with parameters [n,k] over $F_{p^{m}}$. The generator matrix of C is G, and the dimension of the l-Galois hull of C is $\dim({\mathrm{Hull}}_{l}\left(C\right))=h$. Let r=k−h, then there exists a generator matrix $G_{0}$ of C such that*

$$\begin{array}{c} G_{0}\sigma^{l}\left(G_{0}^{T}\right)=\left(\begin{array}{cc} O_{h\times h} & H_{h\times r}\\ O_{r\times h} & P_{r\times r} \end{array}\right), \end{array}$$

*where $O_{h\times h}$ and $O_{r\times h}$ are zero matrices of size h×h and r×h, respectively, and the rank of*

$$\begin{array}{c} Q=\left(\begin{array}{c} H_{h\times r}\\ P_{r\times r} \end{array}\right) \end{array}$$

*is rank(Q)=r. Furthermore, for any generator matrix G of C, the rank of $G\sigma^{l}\left(G^{T}\right)$ is $\mathrm{rank}(G\sigma^{l}\left(G^{T}\right))=r$.*

**Theorem** **4.** *Let $C=e_{1}C_{1}⊕e_{2}C_{2}⊕\cdots ⊕e_{t}C_{t}$ be an $\left(e_{1}\lambda_{1}+e_{2}\lambda_{2}+\cdots +e_{t}\lambda_{t}\right)$-constacyclic code with parameters [n,k] over R. The generator matrix of C is*

$$\begin{array}{c} G=\left(\begin{array}{c} e_{1}G_{1}\\ e_{2}G_{2}\\ \cdots \\ e_{t}G_{t} \end{array}\right), \end{array}$$

*where $C_{i}$ is a $\lambda_{i}$-constacyclic code with parameters $\left[n,k_{i}\right]$, $G_{i}$ is the generator matrix of $C_{i}$. The dimension of the l-Galois hull of $C_{i}$ is $h_{i}$, and $r_{i}=k_{i}-h_{i}$, i=1,2,⋯,t. Then*

$$\begin{array}{c} G\sigma^{l}\left(G^{T}\right)=\left(\begin{array}{cccc} e_{1}G_{1}\sigma^{l}\left(G_{1}^{T}\right) & O & \cdots & O\\ O & e_{2}G_{2}\sigma^{l}\left(G_{2}^{T}\right) & \cdots & O\\ \cdots & \cdots & \cdots & \cdots \\ O & O & \cdots & e_{t}G_{t}\sigma^{l}\left(G_{t}^{T}\right) \end{array}\right) \end{array}$$

*and the rank of $G\sigma^{l}\left(G^{T}\right)$ is $\mathrm{rank}\left(G\sigma^{l}\left(G^{T}\right)\right)=r_{1}+r_{2}+\cdots +r_{t}$.*

**Proof.**  By Lemma 6,

$$\begin{array}{cc} G\sigma^{l}\left(G^{T}\right) & =\left(\begin{array}{c} e_{1}G_{1}\\ e_{2}G_{2}\\ \cdots \\ e_{t}G_{t} \end{array}\right)\sigma^{l}\left(\begin{array}{cccc} e_{1}G_{1}^{T} & e_{2}G_{2}^{T} & \cdots & e_{t}G_{t}^{T} \end{array}\right)\\ & =\left(\begin{array}{c} e_{1}G_{1}\\ e_{2}G_{2}\\ \cdots \\ e_{t}G_{t} \end{array}\right)\left(\begin{array}{cccc} e_{1}\sigma^{l}\left(G_{1}^{T}\right) & e_{2}\sigma^{l}\left(G_{2}^{T}\right) & \cdots & e_{t}\sigma^{l}\left(G_{t}^{T}\right) \end{array}\right)\\ & =\left(\begin{array}{cccc} e_{1}G_{1}\sigma^{l}\left(G_{1}^{T}\right) & O & \cdots & O\\ O & e_{2}G_{2}\sigma^{l}\left(G_{2}^{T}\right) & \cdots & O\\ \cdots & \cdots & \cdots & \cdots \\ O & O & \cdots & e_{t}G_{t}\sigma^{l}\left(G_{t}^{T}\right) \end{array}\right), \end{array}$$

and for i=1,2,⋯,t, $\mathrm{rank}\left(e_{i}G_{i}\sigma^{l}\left(G_{i}^{T}\right)\right)=\mathrm{rank}\left(G_{i}\sigma^{l}\left(G_{i}^{T}\right)\right)=r_{i}$.

So the rank of $G\sigma^{l}\left(G^{T}\right)$ is $\mathrm{rank}\left(G\sigma^{l}\left(G^{T}\right)\right)=r_{1}+r_{2}+\cdots +r_{t}$. □

**Lemma** **7** ([15])**.** *Let C be a linear code with parameters [n,k] over $F_{p^{m}}$, the generator matrix of C is G. Then $\dim\left({\mathrm{Hull}}_{l}\left(C\right)\right)=k-\mathrm{rank}\left(G\sigma^{l}\left(G^{T}\right)\right)$.*

**Lemma** **8.** Let C=〈g(x)〉 be a λ-constacyclic code with parameters [n,k] over $F_{p^{m}}$. Then C is an l-Galois self-orthogonal code if and only if $f\left(x\right)\sigma^{m-l}\left(f^{*}\left(x\right)\right)$ divides $x^{n}-\lambda$, where λ=±1, $f\left(x\right)=\frac{x^{n}-\lambda }{g(x)}$ is the check polynomial of C, and $f^{*}\left(x\right)$ is the reciprocal polynomial of f(x).

**Proof.**  By the same method of Theorem 4 in [26], we have

$$C^{⊥}=\langle f^{*}\left(x\right)\rangle .$$

By Lemma 3, we have

$$\begin{array}{c} C^{{⊥}_{l}}={\left(\sigma \right)}^{m-l}\left(C^{⊥}\right)=\langle \sigma^{m-l}\left(f^{*}\left(x\right)\right)\rangle . \end{array}$$

Hence, ${\mathrm{Hull}}_{l}\left(C\right)=C$ if and only if $C\subseteq C^{{⊥}_{l}}$ if and only if $\exists \;h\left(x\right)\in F_{p^{m}}\left[x\right]$ such that $\sigma^{m-l}\left(f^{*}\left(x\right)\right)h\left(x\right)=g\left(x\right)$ if and only if $f\left(x\right)\sigma^{m-l}\left(f^{*}\left(x\right)\right)h\left(x\right)=f\left(x\right)g\left(x\right)=\left(x^{n}-\lambda \right)$ if and only if $f\left(x\right)\sigma^{m-l}\left(f^{*}\left(x\right)\right)$ divides $x^{n}-\lambda$. □

**Lemma** **9.** *Let $C=e_{1}C_{1}⊕e_{2}C_{2}⊕\cdots ⊕e_{t}C_{t}$ and $D=e_{1}D_{1}⊕e_{2}D_{2}⊕\cdots ⊕e_{t}D_{t}$ be linear codes of length n over R. Then $C\cap D=e_{1}\left(C_{1}\cap D_{1}\right)⊕e_{2}\left(C_{2}\cap D_{2}\right)⊕\cdots ⊕e_{t}\left(C_{t}\cap D_{t}\right)$.*

**Proof.**  For any r∈C∩D, we have

$$\begin{array}{c} r=e_{1}r_{1}+e_{2}r_{2}+\cdots +e_{t}r_{t}\in C=e_{1}C_{1}⊕e_{2}C_{2}⊕\cdots ⊕e_{t}C_{t} \end{array}$$

and

$$\begin{array}{c} r=e_{1}r_{1}+e_{2}r_{2}+\cdots +e_{t}r_{t}\in D=e_{1}D_{1}⊕e_{2}D_{2}⊕\cdots ⊕e_{t}D_{t}, \end{array}$$

which implies

$$\begin{array}{c} r_{i}\in C_{i}\cap D_{i}, \end{array}$$

where i=1,2,⋯,t.

Hence,

$$\begin{array}{c} r\in e_{1}\left(C_{1}\cap D_{1}\right)⊕e_{2}\left(C_{2}\cap D_{2}\right)⊕\cdots ⊕e_{t}\left(C_{t}\cap D_{t}\right). \end{array}$$

Thus

$$\begin{array}{c} C\cap D\subseteq e_{1}\left(C_{1}\cap D_{1}\right)⊕e_{2}\left(C_{2}\cap D_{2}\right)⊕\cdots ⊕e_{t}\left(C_{t}\cap D_{t}\right). \end{array}$$

Conversely, for any

$$\begin{array}{c} \tilde{r}\in e_{1}\left(C_{1}\cap D_{1}\right)⊕e_{2}\left(C_{2}\cap D_{2}\right)⊕\cdots ⊕e_{t}\left(C_{t}\cap D_{t}\right), \end{array}$$

we have

$$\begin{array}{c} \tilde{r}=e_{1}\tilde{r_{1}}+e_{2}\tilde{r_{2}}+\cdots +e_{t}\tilde{r_{t}}, \end{array}$$

which implies

$$\begin{array}{c} \tilde{r}=e_{1}\tilde{r_{1}}+e_{2}\tilde{r_{2}}+\cdots +e_{t}\tilde{r_{t}}\in C \end{array}$$

and

$$\begin{array}{c} \tilde{r}=e_{1}\tilde{r_{1}}+e_{2}\tilde{r_{2}}+\cdots +e_{t}\tilde{r_{t}}\in D, \end{array}$$

where $\tilde{r_{i}}\in C_{i}\cap D_{i}$.

Hence,

$$\begin{array}{c} \tilde{r}\in C\cap D. \end{array}$$

Thus

$$\begin{array}{c} e_{1}\left(C_{1}\cap D_{1}\right)⊕e_{2}\left(C_{2}\cap D_{2}\right)⊕\cdots ⊕e_{t}\left(C_{t}\cap D_{t}\right)\subseteq C\cap D. \end{array}$$

Therefore,

$$\begin{array}{c} C\cap D=e_{1}\left(C_{1}\cap D_{1}\right)⊕e_{2}\left(C_{2}\cap D_{2}\right)⊕\cdots ⊕e_{t}\left(C_{t}\cap D_{t}\right). \end{array}$$

□

**Corollary** **2.** *Let $C=e_{1}C_{1}⊕e_{2}C_{2}⊕\cdots ⊕e_{t}C_{t}$ be an $\left(e_{1}\lambda_{1}+e_{2}\lambda_{2}+\cdots +e_{t}\lambda_{t}\right)$-constacyclic code of length n over R. Then ${\mathrm{Hull}}_{l}\left(C\right)=C$ if and only if ${\mathrm{Hull}}_{l}\left(C_{i}\right)=C_{i}$; and ${\mathrm{Hull}}_{l}\left(C\right)=C^{{⊥}_{l}}$ if and only if ${\mathrm{Hull}}_{l}\left(C_{i}\right)=C_{i}^{{⊥}_{l}}$, where i=1,2,⋯,t.*

**Proof.**  By Lemma 9,

$$\begin{array}{c} \begin{array}{cc} {\mathrm{Hull}}_{l}\left(C\right) & =C\cap C^{{⊥}_{l}}\\ & =e_{1}\left(C_{1}\cap C_{1}^{{⊥}_{l}}\right)⊕e_{2}\left(C_{2}\cap C_{2}^{{⊥}_{l}}\right)⊕\cdots ⊕e_{t}\left(C_{t}\cap C_{t}^{{⊥}_{l}}\right)\\ & =e_{1}{\mathrm{Hull}}_{l}\left(C_{1}\right)⊕e_{2}{\mathrm{Hull}}_{l}\left(C_{2}\right)⊕\cdots ⊕e_{t}{\mathrm{Hull}}_{l}\left(C_{t}\right), \end{array} \end{array}$$

which implies that ${\mathrm{Hull}}_{l}\left(C\right)=C$ if and only if ${\mathrm{Hull}}_{l}\left(C_{i}\right)=C_{i}$, where i=1,2,⋯,t.

By Theorem 1,

$$C^{{⊥}_{l}}=e_{1}C_{1}^{{⊥}_{l}}⊕e_{2}C_{2}^{{⊥}_{l}}⊕\cdots ⊕e_{t}C_{t}^{{⊥}_{l}}.$$

Then we have ${\mathrm{Hull}}_{l}\left(C\right)=C^{{⊥}_{l}}$ if and only if ${\mathrm{Hull}}_{l}\left(C_{i}\right)=C_{i}^{{⊥}_{l}}$, where i=1,2,⋯,t. □

By Corollary 2 and Lemma 8, we can have the following theorem.

**Theorem** **5.** Let $C=e_{1}C_{1}⊕e_{2}C_{2}⊕\cdots ⊕e_{t}C_{t}$ be an $\left(e_{1}\lambda_{1}+e_{2}\lambda_{2}+\cdots +e_{t}\lambda_{t}\right)$-constacyclic code of length n over R. Then ${\mathrm{Hull}}_{l}\left(C\right)=C$ if and only if $f_{i}\left(x\right)\sigma^{m-l}\left(f_{i}^{*}\left(x\right)\right)$ divides $x^{n}-\lambda_{i}$, where $f_{i}\left(x\right)=\frac{x^{n}-\lambda_{i}}{g_{i}\left(x\right)}$ is the check polynomial of $C_{i}$, $f_{i}^{*}\left(x\right)$ is the reciprocal polynomial of $f_{i}\left(x\right)$, i=1,2,⋯,t.

**Lemma** **10** ([9])**.** *Let C=〈p(x)〉 and D=〈q(x)〉 be λ-constacyclic codes of length n over $F_{p^{m}}$, p(x) and q(x) are generator matrices of C and D, respectively. Then C∩D is a λ-constacyclic code generated by lcm(p(x),q(x)).*

By the same method as Theorem 2 in [24], we can have the following theorem.

**Theorem** **6.** *Let $C=e_{1}C_{1}⊕e_{2}C_{2}⊕\cdots ⊕e_{t}C_{t}$ be an $\left(e_{1}\lambda_{1}+e_{2}\lambda_{2}+\cdots +e_{t}\lambda_{t}\right)$-constacyclic code of length n over R, which is generated by $e_{1}g_{1}\left(x\right)+e_{2}g_{2}\left(x\right)+\cdots +e_{t}g_{t}\left(x\right)$. Then*

$$\begin{array}{c} \begin{array}{cc} {\mathrm{Hull}}_{l}\left(C\right) & =C\cap C^{{⊥}_{l}}\\ & =e_{1}\left(C_{1}\cap C_{1}^{{⊥}_{l}}\right)⊕e_{2}\left(C_{2}\cap C_{2}^{{⊥}_{l}}\right)⊕\cdots ⊕e_{t}\left(C_{t}\cap C_{t}^{{⊥}_{l}}\right)\\ & =\langle \sum_{i=1}^{t}e_{i}\mathrm{lcm}\left(g_{i}\left(x\right),\sigma^{m-l}\left(f_{i}^{*}\left(x\right)\right)\right)\rangle , \end{array} \end{array}$$

where $g_{i}\left(x\right)$ is the generator polynomial of $C_{i}$, $f_{i}^{*}\left(x\right)$ is the generator polynomial of $C_{i}^{⊥}$, $\lambda_{i}=\pm 1$, i=1,2,⋯,t.

**Proof.**  By Theorem 2 and Corollary 1,

$$\begin{array}{c} C^{{⊥}_{l}}=e_{1}C_{1}^{{⊥}_{l}}⊕e_{2}C_{2}^{{⊥}_{l}}⊕\cdots ⊕e_{t}C_{t}^{{⊥}_{l}}=\langle \sum_{i=1}^{t}e_{i}\sigma^{m-l}\left(f_{i}^{*}\left(x\right)\right)\rangle \end{array}$$

is an $\left(e_{1}\lambda_{1}+e_{2}\lambda_{2}+\cdots +e_{t}\lambda_{t}\right)$-constacyclic code of length *n* over R.

By Lemma 9,

$$\begin{array}{c} {\mathrm{Hull}}_{l}\left(C\right)=C\cap C^{{⊥}_{l}}=e_{1}\left(C_{1}\cap C_{1}^{{⊥}_{l}}\right)⊕e_{2}\left(C_{2}\cap C_{2}^{{⊥}_{l}}\right)⊕\cdots ⊕e_{t}\left(C_{t}\cap C_{t}^{{⊥}_{l}}\right). \end{array}$$

By Lemma 10, $C_{i}\cap C_{i}^{{⊥}_{l}}$ is a $\lambda_{i}$-constacyclic code generated by $\mathrm{lcm}\left(g_{i}\left(x\right),\sigma^{m-l}\left(f_{i}^{*}\left(x\right)\right)\right)$ for i=1,2,⋯,t.

Thus

$$\begin{array}{c} {\mathrm{Hull}}_{l}\left(C\right)=\langle e_{1}\mathrm{lcm}\left(g_{1}\left(x\right),\sigma^{m-l}\left(f_{1}^{*}\left(x\right)\right)\right),\cdots ,e_{t}\mathrm{lcm}\left(g_{t}\left(x\right),\sigma^{m-l}\left(f_{t}^{*}\left(x\right)\right)\right)\rangle . \end{array}$$

Let

$$\begin{array}{c} \tilde{C}=\langle \sum_{i=1}^{t}e_{i}\mathrm{lcm}\left(g_{i}\left(x\right),\sigma^{m-l}\left(f_{i}^{*}\left(x\right)\right)\right)\rangle , \end{array}$$

which implies that

$$\begin{array}{c} \tilde{C}\subseteq {\mathrm{Hull}}_{l}\left(C\right). \end{array}$$

Then

$$\begin{array}{c} e_{i}\left(\sum_{i=1}^{t}e_{i}\mathrm{lcm}\left(g_{i}\left(x\right),\sigma^{m-l}\left(f_{i}^{*}\left(x\right)\right)\right)\right)=e_{i}\mathrm{lcm}\left(g_{i}\left(x\right),\sigma^{m-l}\left(f_{i}^{*}\left(x\right)\right)\right), \end{array}$$

where i=1,2,⋯,t.

Thus

$$\begin{array}{c} {\mathrm{Hull}}_{l}\left(C\right)\subseteq \tilde{C}. \end{array}$$

Hence,

$$\begin{array}{c} {\mathrm{Hull}}_{l}\left(C\right)=C\cap C^{{⊥}_{l}}=\tilde{C}=\langle \sum_{i=1}^{t}e_{i}\mathrm{lcm}\left(g_{i}\left(x\right),\sigma^{m-l}\left(f_{i}^{*}\left(x\right)\right)\right)\rangle . \end{array}$$

□

**Corollary** **3.** *Let $C=e_{1}C_{1}⊕e_{2}C_{2}⊕\cdots ⊕e_{t}C_{t}$ be a linear code with parameters [n,k] over R, the generator matrix of C is*

$$\begin{array}{c} G=\left(\begin{array}{c} e_{1}G_{1}\\ e_{2}G_{2}\\ \cdots \\ e_{t}G_{t} \end{array}\right), \end{array}$$

*where $G_{i}$ and $k_{i}$ denote the generator matrix and the dimension of $C_{i}$, respectively, i=1,2,⋯,t. Then $\dim\left({\mathrm{Hull}}_{l}\left(C\right)\right)=k-\mathrm{rank}\left(G\sigma^{l}\left(G^{T}\right)\right)$, where $k=k_{1}+k_{2}+\cdots +k_{t}$.*

**Proof.**  By Lemma 9, we have

$$\begin{array}{c} \begin{array}{cc} {\mathrm{Hull}}_{l}\left(C\right) & =C\cap C^{{⊥}_{l}}\\ & =e_{1}\left(C_{1}\cap C_{1}^{{⊥}_{l}}\right)⊕e_{2}\left(C_{2}\cap C_{2}^{{⊥}_{l}}\right)⊕\cdots ⊕e_{t}\left(C_{t}\cap C_{t}^{{⊥}_{l}}\right)\\ & =e_{1}\left({\mathrm{Hull}}_{l}\left(C_{1}\right)\right)⊕e_{2}\left({\mathrm{Hull}}_{l}\left(C_{2}\right)\right)⊕\cdots ⊕e_{t}\left({\mathrm{Hull}}_{l}\left(C_{t}\right)\right), \end{array} \end{array}$$

which implies that

$$\begin{array}{c} \dim\left({\mathrm{Hull}}_{l}\left(C\right)\right)=\dim\left({\mathrm{Hull}}_{l}\left(C_{1}\right)\right)+\dim\left({\mathrm{Hull}}_{l}\left(C_{2}\right)\right)+\cdots +\dim\left({\mathrm{Hull}}_{l}\left(C_{t}\right)\right). \end{array}$$

Let $\dim\left({\mathrm{Hull}}_{l}\left(C_{i}\right)\right)=h_{i}$ and $r_{i}=k_{i}-h_{i}$ for i=1,2,⋯,t. So

$$\begin{array}{c} \dim\left({\mathrm{Hull}}_{l}\left(C\right)\right)=h_{1}+h_{2}+\cdots +h_{t}. \end{array}$$

By Theorem 4,

$$\begin{array}{c} \mathrm{rank}\left(G\sigma^{l}\left(G^{T}\right)\right)=r_{1}+r_{2}+\cdots +r_{t}. \end{array}$$

Hence,

$$\begin{array}{c} \begin{array}{cc} \dim({\mathrm{Hull}}_{l}\left(C\right)) & =h_{1}+h_{2}+\cdots +h_{t}\\ & =k_{1}+k_{2}+\cdots +k_{t}-\left(r_{1}+r_{2}+\cdots +r_{t}\right)\\ & =k-\mathrm{rank}(G\sigma^{l}\left(G^{T}\right)). \end{array} \end{array}$$

□

## 5. Quantum Codes

In this section, we first construct EAQECCs by determining the dimensions of the *l*-Galois hulls of the Gray images of constacyclic codes. Then, we propose a construction X of the Galois inner product by combining the CSS construction, the Hermitian construction, and the construction X, and extend it from $F_{p^{2}}$ to $F_{p^{m}}$. Finally, by applying this construction, we obtain some new quantum codes via the dimensions of the *l*-Galois hulls of constacyclic codes over R.

EAQECC is a type of quantum code that relaxes the dual-containing condition of standard QECC. Formally, a *q*-ary EAQECC, denoted by ${\left[\left[n,k,d;c\right]\right]}_{q}$, encodes *k* information qubits into *n* channel qubits with the help of *c* pairs of maximally entangled ebits, where *d* is the minimum distance, enabling the detection of up to d−1 errors and the correction of up to $\left\lfloor \frac{d-1}{2}\right\rfloor$ errors. Its performance is typically measured by the rate $\frac{k}{n}$ and the net rate $\frac{k-c}{n}$, and it reduces to a standard QECC when c=0. If a QECC with parameters satisfying n−k−2d+2=0 is called QMDS code. Meanwhile, if a QECC with parameters satisfying n−k−2d=0 is called quantum nearly maximum distance separable (QNMDS) code. For more details about EAQECCs, please refer to Refs. [5,6,7,8,16,17,18].

**Theorem** **7.** *Let $C=e_{1}C_{1}⊕e_{2}C_{2}⊕\cdots ⊕e_{t}C_{t}$ be a linear code with parameters [n,k,d] over R. Then ϕ(C) is a linear code with parameters [tn,k,d] and $\phi {\left(C\right)}^{{⊥}_{l}}=\phi \left(C^{{⊥}_{l}}\right)$. If C is an l-Galois self-orthogonal code over R, then ϕ(C) is an l-Galois self-orthogonal code over $F_{p^{m}}$.*

**Proof.**  By Lemma 2, ϕ(C) is a linear code with parameters [tn,k,d].

By the same method of Theorem 5 in [26], we have

$$\begin{array}{c} \phi {\left(C\right)}^{{⊥}_{l}}=\phi \left(C^{{⊥}_{l}}\right). \end{array}$$

If *C* is an *l*-Galois self-orthogonal code, then $C\subseteq C^{{⊥}_{l}}$; consequently,

$$\begin{array}{c} \phi \left(C\right)\subseteq \phi \left(C^{{⊥}_{l}}\right)=\phi {\left(C\right)}^{{⊥}_{l}}. \end{array}$$

Hence, ϕ(C) is an *l*-Galois self-orthogonal code over $F_{p^{m}}$. □

**Corollary** **4.** *Let $C=e_{1}C_{1}⊕e_{2}C_{2}⊕\cdots ⊕e_{t}C_{t}$ be a linear code over R. Then $\phi \left({\mathrm{Hull}}_{l}\left(C\right)\right)={\mathrm{Hull}}_{l}\left(\phi \left(C\right)\right)$.*

**Proof.**  For any $a=\left(a_{1},a_{2},\cdots ,a_{n}\right)\in {\mathrm{Hull}}_{l}\left(C\right)$, where $a_{i}=a_{i,1}e_{1}+a_{i,2}e_{2}+\cdots +a_{i,t}e_{t}$ and $a^{\left(i\right)}=\left(a_{i,1},a_{i,2},\cdots ,a_{i,t}\right)$ for i=1,2,⋯,n, we have $a=(a_{1},a_{2},\cdots ,a_{n})\in C$ and $a=\left(a_{1},a_{2},\cdots ,a_{n}\right)\in C^{{⊥}_{l}}$; moreover, $\phi \left(a\right)=\left(a^{\left(1\right)},a^{\left(2\right)},\cdots ,a^{\left(n\right)}\right)$.

Thus,

$$\begin{array}{c} \begin{array}{cc} 0 & ={\langle a,a\rangle }_{l}\\ & =\sum_{i=1}^{n}\left(e_{1}a_{i,1}a_{i,1}^{p^{l}}+e_{2}a_{i,2}a_{i,2}^{p^{l}}+\cdots +e_{t}a_{i,t}a_{i,t}^{p^{l}}\right)\\ & =e_{1}\sum_{i=1}^{n}a_{i,1}a_{i,1}^{p^{l}}+e_{2}\sum_{i=1}^{n}a_{i,2}a_{i,2}^{p^{l}}+\cdots +e_{t}\sum_{i=1}^{n}a_{i,t}a_{i,t}^{p^{l}}, \end{array} \end{array}$$

which implies $\sum_{i=1}^{n}a_{i,j}a_{i,j}^{p^{l}}=0$ for j=1,2,⋯,t.

Thus,

$$\begin{array}{c} \begin{array}{cc} {\langle \phi \left(a\right),\phi \left(a\right)\rangle }_{l} & ={\langle a^{\left(1\right)},a^{\left(1\right)}\rangle }_{l}+{\langle a^{\left(2\right)},a^{\left(2\right)}\rangle }_{l}+\cdots +{\langle a^{\left(n\right)},a^{\left(n\right)}\rangle }_{l}\\ & =\sum_{j=1}^{t}a_{1,j}a_{1,j}^{p^{l}}+\sum_{j=1}^{t}a_{2,j}a_{2,j}^{p^{l}}+\cdots +\sum_{j=1}^{t}a_{n,j}a_{n,j}^{p^{l}}\\ & =\sum_{i=1}^{n}a_{i,1}a_{i,1}^{p^{l}}+\sum_{i=1}^{n}a_{i,2}a_{i,2}^{p^{l}}+\cdots +\sum_{i=1}^{n}a_{i,t}a_{i,t}^{p^{l}}\\ & =0. \end{array} \end{array}$$

Hence,

$$\begin{array}{c} \phi \left(a\right)\in \phi \left(C\right)\cap \phi {\left(C\right)}^{{⊥}_{l}}={\mathrm{Hull}}_{l}\left(\phi \left(C\right)\right), \end{array}$$

which implies

$$\begin{array}{c} \phi \left({\mathrm{Hull}}_{l}\left(C\right)\right)\subseteq {\mathrm{Hull}}_{l}\left(\phi \left(C\right)\right). \end{array}$$

Conversely, for any

$$\begin{array}{c} \tilde{a}\in {\mathrm{Hull}}_{l}\left(\phi \left(C\right)\right)=\phi \left(C\right)\cap \phi {\left(C\right)}^{{⊥}_{l}}. \end{array}$$

By Theorem 7, we have

$$\begin{array}{c} \phi {\left(C\right)}^{{⊥}_{l}}=\phi \left(C^{{⊥}_{l}}\right) \end{array}$$

and

$$\begin{array}{c} \tilde{a}\in {\mathrm{Hull}}_{l}\left(\phi \left(C\right)\right)=\phi \left(C\right)\cap \phi \left(C^{{⊥}_{l}}\right). \end{array}$$

By the definition of ϕ, there exist x∈C and $y\in C^{{⊥}_{l}}$ such that $\tilde{a}=\phi \left(x\right)=\phi \left(y\right)$.

By Lemma 2, ϕ is a bijection, hence x=y, which implies $x=y\in C\cap C^{{⊥}_{l}}.$

Thus,

$$\begin{array}{c} \tilde{a}=\phi \left(x\right)\in \phi \left(C\cap C^{{⊥}_{l}}\right)=\phi \left({\mathrm{Hull}}_{l}\left(C\right)\right). \end{array}$$

This implies

$$\begin{array}{c} {\mathrm{Hull}}_{l}\left(\phi \left(C\right)\right)\subseteq \phi \left({\mathrm{Hull}}_{l}\left(C\right)\right). \end{array}$$

Therefore,

$$\begin{array}{c} \phi \left({\mathrm{Hull}}_{l}\left(C\right)\right)={\mathrm{Hull}}_{l}\left(\phi \left(C\right)\right). \end{array}$$

□

**Corollary** **5.** *Let $C=e_{1}C_{1}⊕e_{2}C_{2}⊕\cdots ⊕e_{t}C_{t}$ be a linear code over R. Then $\dim\left({\mathrm{Hull}}_{l}\left(\phi \left(C\right)\right)\right)=\dim\left({\mathrm{Hull}}_{l}\left(C\right)\right)$.*

**Proof.**  By Lemma 2, ϕ is a bijection, we have

$$\begin{array}{c} \dim\left({\mathrm{Hull}}_{l}\left(C\right)\right)=\dim\left(\phi ({\mathrm{Hull}}_{l}\left(C\right))\right). \end{array}$$

By Corollary 4, we have

$$\begin{array}{c} \dim\left(\phi ({\mathrm{Hull}}_{l}\left(C\right))\right)=\dim\left({\mathrm{Hull}}_{l}\left(\phi \left(C\right)\right)\right). \end{array}$$

Hence,

$$\begin{array}{c} \dim\left({\mathrm{Hull}}_{l}\left(C\right)\right)=\dim\left(\phi ({\mathrm{Hull}}_{l}\left(C\right))\right)=\dim\left({\mathrm{Hull}}_{l}\left(\phi \left(C\right)\right)\right). \end{array}$$

□

**Lemma** **11** ([16])**.** *Let $C={\left[n,k,d\right]}_{q}$ be a linear code. Then there exists an EAQECC with parameters ${\left[n,k-\dim\left({\mathrm{Hull}}_{l}\left(C\right)\right),d,n-k-\dim\left({\mathrm{Hull}}_{l}\left(C\right)\right)\right]}_{q}$.*

By Theorem 7 and Lemma 11, we can have the following theorem.

**Theorem** **8.** *Let $C=e_{1}C_{1}⊕e_{2}C_{2}⊕\cdots ⊕e_{t}C_{t}$ be a λ-constacyclic code with parameters [n,k,d] over R. Then there exists an EAQECC with parameters ${\left[tn,k-\dim\left({\mathrm{Hull}}_{l}\left(C\right)\right),d,tn-k-\dim\left({\mathrm{Hull}}_{l}\left(C\right)\right)\right]}_{p^{m}}$.*

**Example** **1.** *Let n=15, l=0 and $R=F_{7}\left[u_{1},u_{2}\right]/\langle u_{1}^{2}=u_{1},u_{2}^{2}=u_{2},u_{1}u_{2}=u_{2}u_{1}=0\rangle$. In $F_{7}\left[x\right]$, then $x^{15}+1=\left(x+1\right)\left(x+2\right)\left(x+4\right)\left(x^{4}+3x^{3}+2x^{2}+6x+4\right)\left(x^{4}+5x^{3}+4x^{2}+6x+2\right)\left(x^{4}+6x^{3}+x^{2}+6x+1\right)$ and $x^{15}-1=\left(x+3\right)\left(x+5\right)\left(x+6\right)\left(x^{4}+x^{3}+x^{2}+x+1\right)\left(x^{4}+2x^{3}+4x^{2}+x+2\right)\left(x^{4}+4x^{3}+2x^{2}+x+4\right).$*

*Let C be a $\left(-e_{1}-e_{2}+e_{3}\right)$-constacyclic code of length 15 over R with generator polynomial $e_{1}g_{1}\left(x\right)+e_{2}g_{2}\left(x\right)+e_{3}g_{3}\left(x\right)$, where $g_{1}=x^{4}+3x^{3}+2x^{2}+6x+4$, $g_{2}=x+1$, $g_{3}=x+6$. So ϕ(C) is a linear code with parameters [45,39,4] over $F_{7}$.*

*Hence,*

$$\begin{array}{c} \begin{array}{cc} \dim(\mathrm{Hull}(\phi (C)) & =\dim(\mathrm{Hull}(C))\\ & =\dim\left(\mathrm{Hull}\left(C_{1}\right)\right)+\dim\left(\mathrm{Hull}\left(C_{2}\right)\right)+\dim\left(\mathrm{Hull}\left(C_{3}\right)\right)\\ & =4. \end{array} \end{array}$$

*By Theorem 8, we can get an EAQECC with parameters ${\left[45,35,4,2\right]}_{7}$.*

**Lemma** **12.** *Let $C=e_{1}C_{1}⊕e_{2}C_{2}⊕\cdots ⊕e_{t}C_{t}$ be a linear code over R. Then ${\left(C+C^{{⊥}_{l}}\right)}^{{⊥}_{l}}={\mathrm{Hull}}_{l}\left(C\right)$. Specifically, when l=0 or when m is even and $l=\frac{m}{2}$, then $\left(C+C^{{⊥}_{l}}\right)={\mathrm{Hull}}_{l}{\left(C\right)}^{{⊥}_{l}}$.*

**Proof.**  For any $\;r\in {\left(C+C^{{⊥}_{l}}\right)}^{{⊥}_{l}}$ and any $\;s\in C\subseteq C+C^{{⊥}_{l}}$, we have ${\langle r,s\rangle }_{l}$ = 0, which implies $r\in C^{{⊥}_{l}}$.

For any $\;t\in C^{{⊥}_{l}}\subseteq C+C^{{⊥}_{l}}$, we have ${\langle t,r\rangle }_{l}$=0, which implies r∈C; hence $r\in C\cap C^{{⊥}_{l}}={\mathrm{Hull}}_{l}\left(C\right)$. Thus

$${\left(C+C^{{⊥}_{l}}\right)}^{{⊥}_{l}}\subseteq {\mathrm{Hull}}_{l}\left(C\right).$$

Conversely, for any $\;a\in {\mathrm{Hull}}_{l}\left(C\right)$ and any $b\in C+C^{{⊥}_{l}}$ with $b=c+\overset{´}{c}$ for some c∈C and $\overset{´}{c}\in C^{{⊥}_{l}}$, we have

$$\begin{array}{c} {\langle b,a\rangle }_{l}={\langle c,a\rangle }_{l}+{\langle \overset{´}{c},a\rangle }_{l}=0. \end{array}$$

Thus, $a\in {\left(C+C^{{⊥}_{l}}\right)}^{{⊥}_{l}}$, which implies

$$\begin{array}{c} {\mathrm{Hull}}_{l}\left(C\right)\subseteq {\left(C+C^{{⊥}_{l}}\right)}^{{⊥}_{l}}. \end{array}$$

Hence,

$$\begin{array}{c} {\left(C+C^{{⊥}_{l}}\right)}^{{⊥}_{l}}={\mathrm{Hull}}_{l}\left(C\right). \end{array}$$

Specifically, when l=0 or when *m* is even and $l=\frac{m}{2}$, we have

$$\begin{array}{c} \left(C+C^{{⊥}_{l}}\right)={\left({\left(C+C^{{⊥}_{l}}\right)}^{{⊥}_{l}}\right)}^{{⊥}_{l}}={\mathrm{Hull}}_{l}{\left(C\right)}^{{⊥}_{l}}. \end{array}$$

□

**Lemma** **13.** Let C be a λ-constacyclic code of length n over $F_{p^{m}}$, whose generator polynomial is g(x). Then ${\mathrm{Hull}}_{l}\left(C\right)=C^{{⊥}_{l}}$ if and only if $x^{n}-\lambda$ divides $\sigma^{m-l}\left(f^{*}\left(x\right)\right)f\left(x\right)$, where $f\left(x\right)=\frac{x^{n}-\lambda }{g(x)}$ is the check polynomial of C, $f^{*}\left(x\right)$ is the reciprocal polynomial of f(x), and λ=±1.

**Proof.**  By Lemma 8,

$$C^{⊥}=\langle f^{*}\left(x\right)\rangle .$$

By Lemma 3,

$$\begin{array}{c} C^{{⊥}_{l}}=\sigma^{m-l}\left(C^{⊥}\right)=\langle \sigma^{m-l}\left(f^{*}\left(x\right)\right)\rangle . \end{array}$$

Hence, ${\mathrm{Hull}}_{l}\left(C\right)=C^{{⊥}_{l}}$ if and only if there exists a polynomial $\tilde{f}\left(x\right)\in F_{p^{m}}\left[x\right]$ such that $\sigma^{m-l}\left(f^{*}\left(x\right)\right)=\tilde{f}\left(x\right)g\left(x\right)$, if and only if $\sigma^{m-l}\left(f^{*}\left(x\right)\right)f\left(x\right)=\tilde{f}\left(x\right)g\left(x\right)f\left(x\right)=\tilde{f}\left(x\right)f\left(x\right)g\left(x\right)=\tilde{f}\left(x\right)\left(x^{n}-\lambda \right)$, if and only if $x^{n}-\lambda$ divides $\sigma^{m-l}\left(f^{*}\left(x\right)\right)f\left(x\right)$. □

By Lemma 13 and Corollary 2, we can have the following theorem.

**Theorem** **9.** Let $C=e_{1}C_{1}⊕e_{2}C_{2}⊕\cdots ⊕e_{t}C_{t}$ be a λ-constacyclic code of length n over R. Then ${\mathrm{Hull}}_{l}\left(C\right)=C^{{⊥}_{l}}$ if and only if $x^{n}-\lambda_{i}$ divides $\sigma^{m-l}\left(f^{*}\left(x\right)\right)f_{i}\left(x\right)$, where $\lambda_{i}=\pm 1$, $f_{i}\left(x\right)=\frac{x^{n}-\lambda_{i}}{g_{i}\left(x\right)}$ is the check polynomial of $C_{i}$, and $f_{i}^{*}\left(x\right)$ is the reciprocal polynomial of $f_{i}\left(x\right)$ for i=1,2,⋯,t.

According to the *l*-Galois inner product and the construction X for QECCs over $F_{p^{2}}$ ([20], Theorem 4), we can obtain the construction X of the Galois inner product as follows.

**Theorem** **10** (Construction X of the Galois inner product)**.** *Let C=[n,k,d] be a linear code over $F_{p^{m}}$ and $e=n-k-\dim({\mathrm{Hull}}_{l}\left(C\right))$. If l=0, the l-Galois inner product is the Euclidean inner product; then there exists a QECC with parameters ${\left[\left[n+e,2k-n+e,d\right]\right]}_{p^{m}}$. If m is even and $l=\frac{m}{2}$, the l-Galois inner product is the Hermitian inner product; then there exists a QECC with parameters ${\left[\left[n+e,2k-n+e,d\right]\right]}_{p^{m/2}}$, where $d\geq \min\{d\left(C\right),d\left(C+C^{{⊥}_{l}}\right)+1\}$.*

Clearly, if l=0, it is the onstruction X of the Euclidean inner product. If *m* is even, it is the construction X of the Hermitian inner product. If l=0 and e=0, it is the CSS construction. If *m* is even and e=0, it is the Hermitian construction.

By Theorems 7 and 10, we can have the following theorem.

**Theorem** **11.** *Let $C=e_{1}C_{1}⊕e_{2}C_{2}⊕\cdots ⊕e_{t}C_{t}$ be a λ-constacyclic code of length n with parameters [n,k,d] over R. Then ϕ(C) is a linear code with parameters [tn,k,d] over $F_{p^{m}}$. Let $e=tn-k-\dim({\mathrm{Hull}}_{l}\left(\phi \left(C\right)\right))$. If l=0, then there exists a QECC with parameters ${\left[\left[tn+e,2k-tn+e,d\left(Q\right)\right]\right]}_{p^{m}}$. If m is even and $l=\frac{m}{2}$, then there exists a QECC with parameters ${\left[\left[tn+e,2k-tn+e,d\left(Q\right)\right]\right]}_{p^{m/2}}$, where $d\left(Q\right)\geq \min\{d,d\left(\phi \left(C\right)+{\left(\phi \left(C\right)\right)}^{{⊥}_{l}}\right))+1\}$.*

**Example** **2.** *Let n=8, l=1 and $R=F_{9}\left[u_{1},u_{2}\right]/\langle u_{1}^{2}=u_{1},u_{2}^{2}=u_{2},u_{1}u_{2}=u_{2}u_{1}=0\rangle$. In $F_{9}\left[x\right]$, $x^{8}-1=\left(x+1\right)\left(x+w\right)\left(x+w^{2}\right)\left(x+w^{3}\right)\left(x+2\right)\left(x+w^{5}\right)\left(x+w^{6}\right)\left(x+w^{7}\right).$*

*Let C be an $\left(e_{1}+e_{2}+e_{3}\right)$-constacyclic code of length 8 over R. Let $g_{1}\left(x\right)=\left(x+w\right)\left(x+w^{2}\right)$, $g_{2}\left(x\right)=x+w^{3}$ and $g_{3}\left(x\right)=x+w^{3}$, then $C_{1}=\langle g_{1}\left(x\right)\rangle$, $C_{2}=\langle g_{2}\left(x\right)\rangle$ and $C_{3}=\langle g_{3}\left(x\right)\rangle$ are cyclic codes of length 8 over $F_{9}$.*

*By Theorem 7, ϕ(C) is a linear code over $F_{9}$ with parameters [24,20,3].*

*Hence,*

$$\begin{array}{c} \begin{array}{cc} \dim(\mathrm{Hull}(\phi (C)) & =\dim(\mathrm{Hull}(C))\\ & =\dim\left(\mathrm{Hull}\left(C_{1}\right)\right)+\dim\left(\mathrm{Hull}\left(C_{2}\right)\right)+\dim\left(\mathrm{Hull}\left(C_{3}\right)\right)\\ & =3 \end{array} \end{array}$$

*and*

e=24−20−3=1.

*By Lemma 12,*

$$\begin{array}{c} \phi \left(C\right)+{\left(\phi \left(C\right)\right)}^{⊥}={\left(\mathrm{Hull}\left(\phi \left(C\right)\right)\right)}^{⊥}={\left(\phi \left(\mathrm{Hull}\left(C\right)\right)\right)}^{⊥}=\phi \left({\left(\mathrm{Hull}\left(C\right)\right)}^{⊥}\right) \end{array}$$

*and*

$$\begin{array}{c} d\left(\phi \left(C\right)+{\left(\phi \left(C\right)\right)}^{⊥}\right)=d\left(\phi \left({\left(\mathrm{Hull}\left(C\right)\right)}^{⊥}\right)\right)=d{\left(\mathrm{Hull}\left(C\right)\right)}^{⊥}=d\left(C\right)=3. \end{array}$$

*By Theorem 11, we can get a QECC with parameters ${\left[\left[25,17,\geq 3\right]\right]}_{3}$.*

In Table 1, we provide some new QECCs (in the eighth column) that are not available in the database of quantum codes [28]. Among them, the first three are QMDS codes, while the fourth to ninth are QECCs with better parameters compared to Ref. [28] (they have higher code rates than those in Ref. [28] under the same minimum distance), and the last one is a QECC over $F_{81}$ (Ref. [28] does not have a QECC over $F_{81}$). In particular, the fourth and fifth are QNMDS codes. Moreover, the value of $p^{m}$, the length *n*, the value of *l*, the tuple $\left(l_{1},\ldots ,l_{s}\right)$ of the ring R, and the tuple of units $\left(\lambda_{1},\lambda_{2},\ldots ,\lambda_{t}\right)$ are given in the first five columns, respectively. The generator polynomial $\langle g_{1}\left(x\right),g_{2}\left(x\right),\ldots ,g_{t}\left(x\right)\rangle$ is given in the sixth column, where $g_{i}\left(x\right)=a_{n}x^{n}+a_{n-1}x^{n-1}+\cdots +a_{1}x+a_{0}$ is denoted by $a_{n}a_{n-1}\cdots a_{1}a_{0}$. The seventh column gives the parameters of ϕ(C). In the ninth and eleventh columns, we give the code rates of the new QECCs and the QECCs in Ref. [28], respectively.

## 6. Conclusions

In this article, we construct quantum codes by determining the dimensions of the *l*-Galois hull of constacyclic codes over a finite non-chain ring R. In addition, we propose a generalized Construction X for the Galois inner product and extend it from $F_{p^{2}}$ to $F_{p^{m}}$. By using this framework, we obtain some new QMDS codes, and some new QECCs that are better than the best known ones.

This work provides an extension of Construction X for quantum codes and enriches the source of quantum code constructions. However, it is tailored to the specific ring R, and the extension of Construction X relies on the determinations of the *l*-Galois hull, which is restricted to the case where *l* takes special values.

In future research, we plan to construct quantum codes with better parameters by considering Galois hulls of arbitrary dimensions over other finite rings, such as chain rings or different non-chain rings.

## Figures and Tables

**Table 1:** New QECCs from constacyclic codes over R.

$p^{m}$	*n*	*l*	$\left(l_{1},\cdots ,l_{s}\right)$	$\left(\lambda_{1},\lambda_{2},\cdots ,\lambda_{t}\right)$	$\langle g_{1}\left(x\right),g_{2}\left(x\right),\cdots ,g_{t}\left(x\right)\rangle$	ϕ(C)	New QECCs	Code Rate	QECCs in Ref. [28]	Code Rate
$3^{2}$	30	0	(2)	(1,1)	$\left(1\omega^{2}\omega^{2}1,11\right)$	[60,57,4]	${\left[\left[60,54,4\right]\right]}_{9}$	0.90	MDS	–
$7^{2}$	43	0	(2)	(1,1)	$\left(1\omega^{3}\omega^{45}6,1\right)$	[86,83,4]	${\left[\left[86,80,4\right]\right]}_{49}$	0.93	MDS	–
$13^{2}$	14	0	(2)	(−1,−1)	$\left(1\omega^{21}1,1\omega^{162}\right)$	[28,25,4]	${\left[\left[28,22,4\right]\right]}_{169}$	0.79	MDS	–
3	12	0	(3, 3, 3, 2)	(1,1,1,1,1,1,1,1)	(1111,11,1,1,1,1,1,1)	[96,92,4]	${\left[\left[96,88,4\right]\right]}_{3}$	0.92	${\left[\left[96,86,4\right]\right]}_{3}$	0.90
13	35	0	(2)	(1,1)	(197(11)1,1)	[70,66,4]	${\left[\left[70,62,4\right]\right]}_{13}$	0.89	${\left[\left[36,24,4\right]\right]}_{13}$	0.67
7	28	0	(3, 2)	(1,1,1,1)	(1111,11,11,11)	[112,106,4]	${\left[\left[112,100,4\right]\right]}_{7}$	0.89	${\left[\left[112,92,4\right]\right]}_{7}$	0.82
$3^{2}$	16	0	(3, 2)	(1,1,1,1)	$\left(1\omega^{2}\omega^{3}\omega^{5},1\omega^{2},1\omega^{2},1\omega^{2}\right)$	[64,58,3]	${\left[\left[64,52,3\right]\right]}_{9}$	0.81	${\left[\left[51,39,3\right]\right]}_{9}$	0.76
$5^{2}$	29	0	(2)	(1,1)	$\left(1\omega^{2}\omega^{3}\omega \omega^{17}\omega^{3}\omega^{22}4,1\right)$	[58,51,6]	${\left[\left[58,44,6\right]\right]}_{25}$	0.76	${\left[\left[16,4,6\right]\right]}_{25}$	0.25
$7^{2}$	16	0	(3, 2)	(1,1,1,1)	$\left(1\omega^{7}\omega^{3},1\omega^{3},1\omega^{3},1\omega^{3}\right)$	[64,59,3]	${\left[\left[64,54,3\right]\right]}_{49}$	0.84	${\left[\left[56,40,3\right]\right]}_{49}$	0.71
$3^{4}$	29	0	(2)	(1,1)	$\left(1\omega^{23}\omega^{22}\omega^{48}\omega^{72}\omega^{78}\omega^{7}2,12\right)$	[58,50,7]	${\left[\left[58,42,7\right]\right]}_{81}$	0.72	None	–

## Data Availability

Data is contained within the article.

## Abbreviations

The following abbreviations are used in this manuscript:

CSS	Calderbank–Shor–Steane
QMDS	quantum maximum distance separable
QECCs	quantum error-correcting codes
QECC	quantum error-correcting code
EAQECC	entanglement-assisted quantum error-correcting code
LCD	linear complementary dual codes
MDS	maximum distance separable
QNMDS	quantum nearly maximum distance separable
